# Stated preference data on the insurance value of forests in Switzerland

**DOI:** 10.1016/j.dib.2020.106466

**Published:** 2020-10-27

**Authors:** Christian Unterberger, Roland Olschewski

**Affiliations:** WSL Swiss Federal Institute for Forest, Snow and Landscape Research, Zürcherstrasse 111, CH-8903, Birmensdorf, Switzerland

**Keywords:** Discrete choice, Ecosystem services, Climate change adaptation, Resilience

## Abstract

We present stated preference data for improved forest management from seven Swiss municipalities in the Cantons of Grisons and Valais. The data was collected between October 2019 and February 2020 using an online questionnaire. We invited 10289 households to participate and received 939 responses. The online questionnaire consisted of two main parts – (1) an online choice experiment and (2) questions on the sociodemographic characteristics of the responding households. The choice experiment confronted households with twelve consecutive choice tasks. Each choice task consisted of three options with a varying degree of avalanche and rock fall risk reduction due to improved forest management. The options further differed with respect to the way the charges for the improved forest management are determined and assigned to the households. We additionally included a cost attribute to estimate the respondents' willingness to pay. At the end of the choice experiment we asked five de-briefing questions and eight attitudinal questions. Additionally, we asked the responding households to state their willingness to take risks. The sociodemographic characteristics collected in the second part of the questionnaire allowed us to analyse their impact on the choices we observed in the first part of the questionnaire. An analysis of the choice data and further interpretive insights are presented in the article “Determining the insurance value of ecosystems: A discrete choice study on natural hazard protection by forests”.

**Specifications Table**

**Table:** 

Subject	Economics and Econometrics Environmental Economics
Specific subject area	Valuation of ecosystem services
Type of data	CSV data file
How data were acquired	Online questionnaire Sawtooth
Data format	Raw data
Parameters for data collection	The questionnaire targeted households in seven Swiss municipalities in the Canton of Grisons and Valais, which are exposed to avalanche and rock fall risk.
Description of data collection	Around 10,300 invitation letters were sent to households in seven Swiss municipalities. Each letter contained login details to an online questionnaire, which we administered via Sawtooth. The questionnaire consisted of two parts, a choice experiment and questions on sociodemographic characteristics of the responding households.
Data source location	Davos/ Canton of Grisons/ Switzerland Bergün Filisur/ Canton of Grisons/ Switzerland Albula/Alvra/ Canton of Grisons/ Switzerland St. Niklaus/ Canton of Valais/ Switzerland Grächen/ Canton of Valais/ Switzerland Täsch/ Canton of Valais/ Switzerland Zermatt/ Canton of Valais/ Switzerland
Data accessibility	Data is accessible via EnviDat, the WSL data portal Repository name: EnviDat (https://www.envidat.ch/) Data identification number: https:/doi.org/10.16904/envidat.175 Direct URL to data: https://www.envidat.ch/dataset/stated-preference-data-on-the-insurance-value-of-forests-in-switzerland
Related research article	Unterberger C, Olschewski R. Determining the insurance value of ecosystems: A discrete choice study on natural hazard protection by forests. Ecological Economics. in press

**Value of the Data**

- The data allow for eliciting preferences of households for funding improved forest management that is geared towards reducing avalanche and rock fall risk. Additionally, it helps to see how variations in sociodemographic characteristics affect these preferences. Eventually, the data provide a demand side perspective on the insurance value of ecosystems.
- The data helps to inform policy makers as well as the private sector (insurance companies) when it comes to incorporating ecosystem services into climate change adaptation and disaster risk management agendas.
- The data can be used to operationalize the insurance value of ecosystems. In particular, this dataset enables a demand side perspective on the insurance value of ecosystems and helps to analyze preferences and the willingness to pay based on sociodemographic characteristic.
- Preferences and willingness to pay estimates on the demand side can be linked with insights from the supply side (the provisioning of ecosystem services) in order to identify feasible strategies and policies.

## 1 Data Description

We conducted a discrete choice study to elicit the preferences of Swiss households for forest management that is geared towards the reduction of natural hazards. We focused on seven municipalities in the Cantons of Grisons and Valais. In each of these municipalities, households face avalanche and rock fall risks. The first part of our online questionnaire consisted of a discrete choice study in which respondents face 12 consecutive choice tasks. In each choice task, respondents are asked to choose one among three alternatives. Each of these alternatives had five attributes with two to six attribute levels. The attributes and their respective levels are presented in Table 1 in Section 2.1. After the choice experiment, we asked five de-briefing questions as shown in Table 2 in Section 2.1, followed by eight attitudinal questions as shown in Table 3. In the second part of the survey, we asked respondents about their sociodemographic characteristics, e.g., age, gender, monthly disposable income per household, type of residence, property situation etc. The questions on the sociodemographic characteristics are shown in Table 4 in Section 2.2 below. Following [1,2], we additionally asked respondents to state their willingness to take risks on a scale ranging from 0 to 10, with 0 referring to *“completely unwilling to take risks”* and 10 to *“very willing to take risks”*. The here presented data is thus a combination of the choices respondents made within the choice experiment and their stated sociodemographic characteristics. For reasons of confidentiality we anonymized the data by removing all fields that would enable personal identification (IP-address, email address, comments, password, username). The complete questionnaire, the dataset and data description are available on the Environmental Data Platform EnviDat of the Swiss Federal Institute for Forest, Snow and Landscape Research WSL (doi:10.16904/envidat.175). “DIVES_Questionnaire.pdf” shows the complete questionnaire. “DIVES_choice_data.csv” is the dataset and “data_description.pdf” provides its detailed description.

**Table 1:** Attributes and their respective attribute levels (readapted from [3]).

Attribute	Levels
Hazard zone	Red / Blue / White
Protection extended to traffic infrastructure	Yes / No
Costing method	Risk based / Lump sum
Contribution mode	Voluntary / Mandatory
Additional annual charge per household	CHF 0 / 100 / 300 / 500 / 700 / 900

**Table 2:** De-briefing questions.

	Strongly disagree	Rather not agree	Partly/ partly	Rather agree	Strongly agree	Do not know/ not specified
The questions were phrased in an understandable way	Ο	Ο	Ο	Ο	Ο	Ο
I felt safe making the decisions	Ο	Ο	Ο	Ο	Ο	Ο
I felt urged to give certain answers	Ο	Ο	Ο	Ο	Ο	Ο
The choice situations were realistic	Ο	Ο	Ο	Ο	Ο	Ο
I consider the annual charge per household realistic	Ο	Ο	Ο	Ο	Ο	Ο

**Table 3:** Attitudinal questions.

	Doesn't apply at all	Rather does not apply	Partly/ partly	Rather applies	Fully applies	Do not know/ not specified
I am well familiar with the protective impact of the forest in my municipality.	Ο	Ο	Ο	Ο	Ο	Ο
My own exposure to avalanches and rock fall is low.	Ο	Ο	Ο	Ο	Ο	Ο
The current silvicultural measures for avalanche and rock fall protection in my						
municipality are sufficient.	Ο	Ο	Ο	Ο	Ο	Ο
Over the next 50 years, climate change will increasingly contribute to wind throw and						
pest infestation in forests.	Ο	Ο	Ο	Ο	Ο	Ο
Over the next 50 years, climate change will lead to a sharp decline in avalanches and						
rock fall hazards.	Ο	Ο	Ο	Ο	Ο	Ο
Avalanche protection is the sole responsibility of the federal and cantonal authorities.	Ο	Ο	Ο	Ο	Ο	Ο
Climate change is aggravating the natural hazard situation.	Ο	Ο	Ο	Ο	Ο	Ο
I am generally concerned about the effects of climate change.	Ο	Ο	Ο	Ο	Ο	Ο

**Table 4:** Questions on the sociodemographic characteristics of the responding households.

Sociodemographic characteristics	Type of question	Answer options
Gender	Select	- Male - Female - Other
Year of birth	Input field	Numeric YYYY
Monthly household income	Select	- Less than CHF 2000 - CHF 2000 to below CHF 4000 - CHF 4000 to below CHF 6000 - CHF 6000 to below CHF 8000 - CHF 8000 to below CHF 10000 - CHF 10000 to below CHF 12000 - CHF 12000 to below CHF 14000 - CHF 14000 to below CHF 16000 - CHF 16000 to below CHF 18000 - More than CHF 18000
What kind of building do you live in?	Select	- Single-family house - Multi-family house - Other
Do you own or rent a house or apartment in this municipality?	Select	- Owner - Tenant
How many people live in your household?	Input filed	Numeric
How many children under 18 live in your household?	Select	- 0 - 1 - 2 - 3 and more
For how many years have you been living in your present house or apartment?	Input field	Numeric
For how many years have you been living in this municipality?	Input field	Numeric
In which hazard zone do you actually live?	Select	- In the red hazard zone, - In the blue hazard zone, - In the white hazard zone, - I don't know
What is your highest educational qualification?	Select	- No graduation - Primary, secondary, secondary modern, or district school - Apprenticeship, vocational school or commercial school - A-levels, vocational school-leaving certificate, diploma from an intermediate diploma, or teacher seminar - Swiss federal vocational diploma, degree from a higher education institution, - Master craftsman's examination, or commercial college - University, ETH, or university of applied sciences
Are you or a person in your household a member of a non-profit club or association?	Select	- Yes - No

## 2 Experimental Design, Materials and Methods

In October 2019 we sent invitation letters to all households in our seven case study municipalities in the Cantons of Grisons, GR, and Valais, VS (VS: St. Niklaus, Grächen, Täsch, Zermatt, GR: Davos, Albula/Alvra/Bergün Filisur). For the households in GR and the municipality of Täsch, we received the address data of all registered households from the municipal authorities. Due to the large number of households in Davos we only invited households where the main person registered was born in an even year. To contact the households in Zermatt, Grächen and St. Niklaus we used bulk mailing. Overall, we invited 10,289 households. The online questionnaire was open between October 2019 and February 2020. In these five months we received 939 responses. We administered the online questionnaire with the hosting service provided by Sawtooth. For the layout of the questionnaire we used Sawtooth's survey software Lighthouse Studio.

The questionnaire consisted of two main parts: (1) A choice experiment with twelve consecutive choice tasks and the associated de-briefing and attitudinal questions. (2) Questions on the sociodemographic characteristics of the household.

### 2.1 The choice experiment

The choice experiment consisted of twelve consecutive choice tasks. In each choice task respondents had to choose between three alternatives. Options 1 and 2 described situations in which better forest management leads to reduced avalanche and rock fall risk. The status quo in each choice task described a situation without improved forest management, hence there is no change in the avalanche and rock fall risk faced. To identify relevant attributes and credible attribute levels we collaborated with insurance and re-insurance professionals and academics from different disciplines. In the course of workshops and project meetings we identified five relevant attributes and the respective levels. These are shown in Table 1.

We used Ngene to generate a D-efficient design that varies the attribute levels in Options 1 and 2 and has a constant status quo across twelve choice tasks [4]. For a detailed description of the attribute levels and the choice experiment please refer to the accompanying publication [3].

Subsequent to the choice experiment we asked the responding household five de-briefing questions to understand whether the households understood the experiment and considered it realistic. For each question the possible response ranged from “strongly disagree” to “strongly agree” with the opportunity not to state any answer. Table 2 below shows the debriefing questions and the possible answers.

Next, we asked eight attitudinal questions to get an idea of the subjective risk exposure of the responding households as well as subjective valuations and opinions on natural hazards, forest management and their link to climate change. They are shown in Table 3.

Finally, we asked the responding household to indicate their willingness to take risks. Following [1,2], we used a scale ranging from 0 to 10, with 0 referring to *“absolutely no willingness to take risks”* and 10 to *“very high willingness to take risks”*.

### 2.2 Sociodemographic characteristics

In the second part of the questionnaire we asked the responding households to share some sociodemographic characteristics. The questions and the respective answer options are presented in Table 4.

## Ethics Statement

The participation in the survey was voluntary and respondents were informed that the data will be analyzed anonymously. In participating in the survey and submitting the questionnaire each respondent gave her/his informed consent.

## Declaration of Competing Interest

The authors declare that they have no known competing financial interests or personal relationships which have, or could be perceived to have, influenced the work reported in this article.
